# Severe HCoV‐OC43 Pneumonia in a 25‐Year‐Old Woman Following Allogeneic Hematopoietic Stem Cell Transplantation

**DOI:** 10.1111/crj.70196

**Published:** 2026-05-11

**Authors:** Mingzhou Zhang, Ting Tang, Mingxia Zheng, Menglin Luo, Zhi Xu, Guansong Wang

**Affiliations:** ^1^ Department of Respiratory and Critical Care Medicine Xinqiao Hospital of Third Military Medical University (Army Medical University) Chongqing China; ^2^ Chongqing Key Laboratory of Precision Medicine and Prevention of Major Respiratory Diseases Chongqing China; ^3^ Department of Respiratory Disease Chongqing Public Health Emergency Medical Center Chongqing China; ^4^ Department of Pharmacy Xinqiao Hospital, Third Military Medical University Chongqing China

**Keywords:** HCoV‐OC43, nirmatrelvir, pneumonia, ritonavir, stem cell transplantation, viral infection

## Abstract

**Background:**

We report the case of a 25‐year‐old woman with a history of acute lymphoblastic leukemia who developed progressive dyspnea and fever following allogeneic hematopoietic stem cell transplantation.

**Case Presentation:**

Despite broad‐spectrum empirical antimicrobial therapy, the patient's clinical condition worsened. Comprehensive diagnostic evaluation revealed human coronavirus OC43 (HCoV‐OC43) as the causative pathogen. Histopathological examination of lung tissue confirmed viral pneumonia. Treatment with nirmatrelvir/ritonavir (Paxlovid) was initiated. The patient demonstrated rapid clinical improvement, with complete radiological resolution within 3 months.

**Conclusion:**

This case highlights the potential therapeutic role of nirmatrelvir/ritonavir in managing severe HCoV‐OC43 pneumonia in patients who are immunocompromised.

## Graphical abstract

This case reports a 25‐year‐old allo‐HCT recipient with severe HCoV‐OC43 pneumonia who responded well to off‐label nirmatrelvir/ritonavir. It is the first such report that show the drug may benefit immunocompromised patients with this infection, though further studies are needed to confirm efficacy.

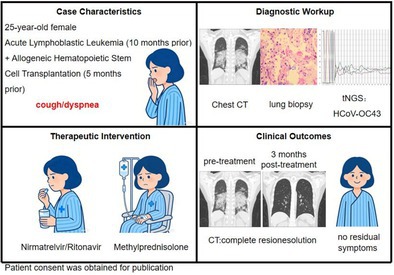

## 1 Introduction

Human coronavirus OC43 (HCoV‐OC43) predominantly affects children aged < 5 years [1], causing mild acute upper respiratory tract infections resembling the common cold [2]. However, sporadic cases of severe or fatal infection have been reported in older adults and immunocompromised individuals. To date, no targeted antiviral therapies have been approved for HCoV‐OC43 infection. Herein, we describe a case of severe HCoV‐OC43 pneumonia treated with nirmatrelvir/ritonavir (Paxlovid), with the aim of providing clinical insights for managing similar cases.

## 2 Case Presentation

A 25‐year‐old woman with acute lymphoblastic leukemia (diagnosed 10 months earlier) who had undergone allogeneic hematopoietic stem cell transplantation (allo‐HCT) 5 months earlier presented with a 2‐week history of low‐grade fever (maximum temperature 37.8°C), cough, and progressive dyspnea. She was receiving cyclosporine for immunosuppression (10 mg in the morning and 20 mg in the evening for the past 2 months, without concurrent corticosteroid therapy) and cotrimoxazole prophylaxis and had not developed graft‐versus‐host disease (GVHD). The cyclosporine trough concentration was within the therapeutic range (198 ng/mL; target range, 150–250 ng/mL).

The patient reported that her symptoms began after exposure to cold. Despite oral ibuprofen for symptomatic relief, the fever persisted. One week before admission, cough and dyspnea developed and progressively worsened.

The patient initially received 72‐h empiric therapy with piperacillin–tazobactam, vancomycin, and sulfamethoxazole–trimethoprim, during which cyclosporine was temporarily discontinued; however, fever and hypoxemia worsened, prompting hospital admission. Upon hospitalization, treatment was escalated to include posaconazole for possible fungal infection [3], amoxicillin–clavulanate for bacterial coverage, and sulfamethoxazole for *Pneumocystis carinii* prophylaxis.

On admission, her body temperature was 36.3°C, heart rate 117 bpm, blood pressure 97/61 mmHg, and peripheral oxygen saturation 90% on room air. The PaO2/FiO2 ratio was 290 mmHg, consistent with mild acute respiratory distress. Lung auscultation revealed scattered fine crackles at the bases, whereas the remainder of the physical examination was unremarkable.

**FIGURE 1:**
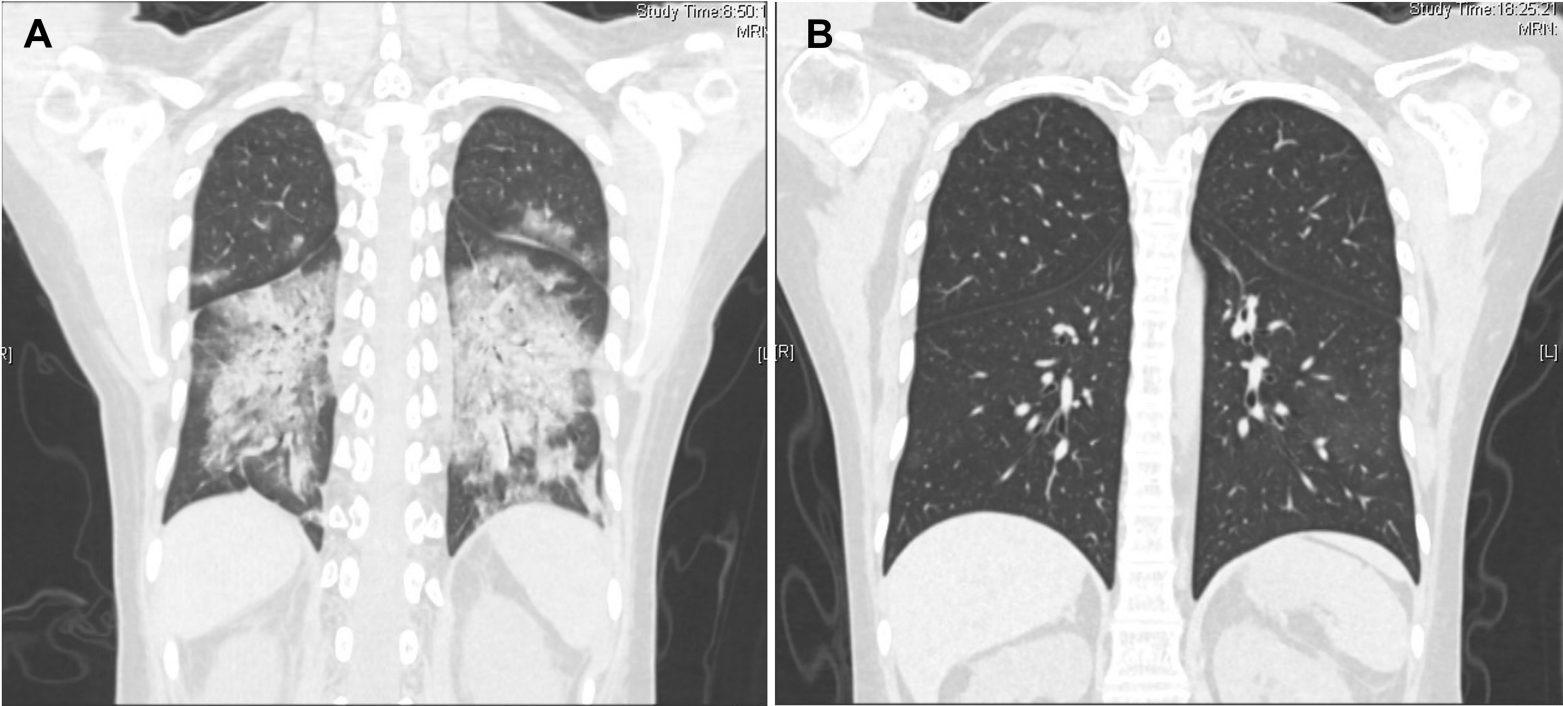
(A) Chest computed tomography (CT) scan obtained on admission showing multiple patchy and streaky areas of increased attenuation in both lungs, accompanied by bilateral ground‐glass opacities. (B) Follow‐up chest CT scan at 3 months demonstrating complete radiological resolution of the pulmonary lesions.

**FIGURE 2:**
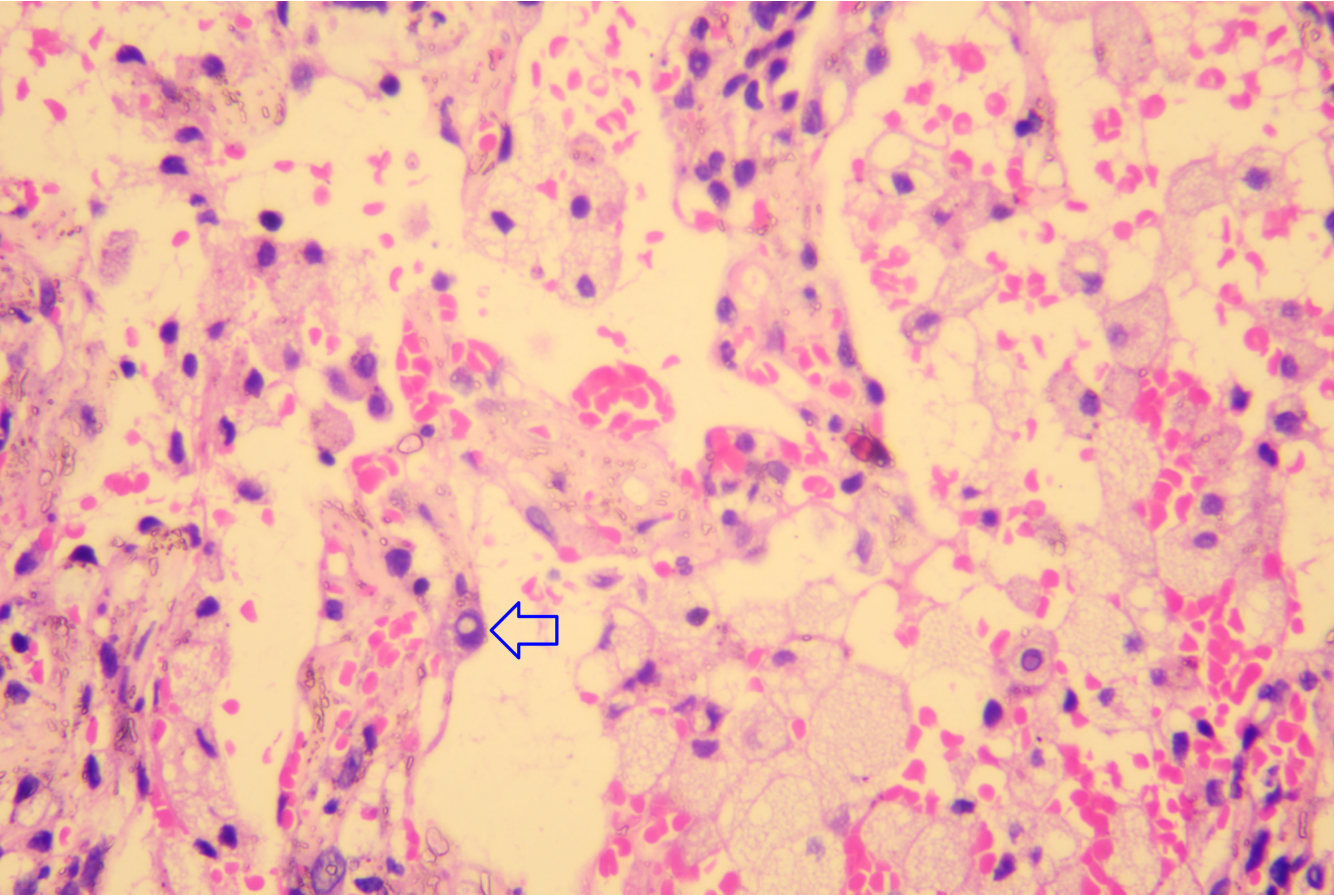
Histopathological examination of lung tissue showing alveolar oedema with red blood cells and detached alveolar epithelial cells within the alveolar spaces. Prominent fibroblast proliferation and lymphocytic infiltration of the pulmonary interstitium, as well as inflammatory cell infiltration of the bronchial submucosa, can be observed. Notably, translucent inclusion bodies approximately the size of red blood cells are present within alveolar epithelial cells (blue arrow). Original magnification, 400×.

Laboratory investigations showed a normal C‐reactive protein level (< 5 mg/L), neutropenia (neutrophil count: 2.93 × 109/L; reference: 4.5–10.5 × 109/L), IgG (4.14 g/L; target range, 7.0–16.0 g/L), lymphopenia (lymphocyte count: 0.69 × 109/L; reference: 0.8–3.5 × 109/L), CD4+ (43.75 cells/μL; target range, 500–1400 cells/μL), CD4+/CD8 + 0.14, and anemia (hemoglobin level: 9.7 g/dL; reference: 13.0–17.0 g/dL). Liver and renal function tests were normal. Routine microbiological investigations, including sputum culture, tuberculosis screening, and HIV serology, were negative. Chest computed tomography (CT) demonstrated exudative lesions with multiple patchy areas of increased density and bilateral ground‐glass opacities consistent with pneumonia symptoms (Figure 1A). Upper respiratory multiplex PCR panel (BioFire FilmArray Respiratory Panel 2.1) obtained on admission was negative for influenza A/B, respiratory syncytial virus, human metapneumovirus, parainfluenza viruses, rhinovirus/enterovirus, adenovirus, and bocavirus. Bronchoscopy was performed, and transbronchial lung biopsy specimens were obtained for histopathology. Targeted next‐generation sequencing of bronchoalveolar lavage fluid (BALF) revealed 245 079 sequence reads of HCoV‐OC43, whereas screening for other pathogens was negative; BALF bacterial culture, fungal culture, acid‐fast bacilli smear, and tuberculosis PCR (Xpert MTB/RIF) were all negative. Targeted next‐generation sequencing of BALF revealed no other pathogenic sequences detected above the reporting threshold (viral reads > 100 or bacterial reads > 500). Histopathology revealed viral inclusions, fibroblast proliferation, interstitial lymphocytic infiltration, and edema, consistent with viral pneumonia manifestations (Figure 2) [4, 5].

Antiviral therapy was initiated with oral nirmatrelvir (300 mg) with ritonavir (100 mg) every 12 h. Intravenous methylprednisolone (40 mg daily) was also administered to control inflammation. Within 6 h of treatment initiation, fever resolved and dyspnea improved slightly. By Day 3, nocturnal cough frequency had decreased, although exertional dyspnea persisted. Mild crackles remained audible over the left lung, and the PaO_2_/FiO_2_ ratio was 256 mmHg, consistent with mild respiratory distress. By Day 5, both cough and dyspnea had markedly improved, and the PaO_2_/FiO_2_ ratio increased to 320 mmHg, allowing discontinuation of supplemental oxygen.

Nirmatrelvir/ritonavir was discontinued on Day 6, and corticosteroids were tapered over the subsequent 5 days. A repeat chest CT performed on Day 10 showed significant resolution of the exudative lesions. No significant adverse events occurred during treatment, and alanine aminotransferase, aspartate aminotransferase, and serum creatinine levels remained within normal limits. At the 4‐week follow‐up, the patient reported occasional cough. At the 3‐month follow‐up, she was asymptomatic, and chest CT showed complete radiological resolution of pulmonary lesions (Figure 1B).

## 3 Discussion

HCoV‐OC43 was first identified in the 1960s using tracheal explant cultures, from which its designation “OC” for “organ culture” was derived. In immunocompetent hosts, it typically causes mild upper respiratory tract infection resembling the common cold and is associated with low hospitalization rates, reflecting its generally benign clinical course. Nevertheless, increasing evidence indicates that HCoV‐OC43 can cause severe and occasionally fatal lower respiratory tract infections, particularly in older adults and immunocompromised individuals, including children with acute lymphoblastic leukemia [6, 7, 8, 9]. These observations underscore the potential severity of this virus in vulnerable populations.

Seasonal coronaviruses (HCoV‐229E, HCoV‐OC43, HCoV‐NL63, and HCoV‐HKU1) are increasingly recognized as important causes of severe pneumonia in allo‐HCT recipients. In a prospective cohort study in the United States, HCoV infection was detected in 4.8% of transplant recipients within 100 days posttransplantation, with 1.1% developing lower respiratory tract disease and a 30‐day mortality rate of 18% [8]. Similarly, Ogimi et al. [9] reported a 6‐month cumulative incidence of 3.2% for HCoV pneumonia in allo‐HCT recipients, with a case‐fatality rate of 26%. Independent risk factors for poor outcomes included cord blood transplantation, profound lymphopenia (< 200/μL), and high‐dose systemic corticosteroid exposure (≥ 2 mg/kg/day). High viral load in BALF is also associated with increased mortality.

Our patient had a history of acute lymphoblastic leukemia and had undergone allo‐HCT 5 months prior to presentation. The patient was receiving cyclosporine for GVHD prophylaxis, resulting in sustained immunosuppression and heightened susceptibility to infection. Although bacterial and fungal infections predominate during the early posttransplant period, particularly within the first 100 days [3], viral infections become increasingly prevalent as immune reconstitution progresses. Consequently, this patient was at high risk for severe viral lower respiratory tract infection, including HCoV‐OC43 pneumonia [1, 2, 6, 8, 9]. Secondary bacterial or fungal pneumonia following respiratory viral infection is typically marked by declining viral loads and a shift towards neutrophil‐predominant inflammation, as shown in both experimental models and clinical cohorts [10, 11]. In contrast, progressive primary viral pneumonia in the absence of effective viral clearance is characterized by persistently high viral RNA levels and predominantly lymphocytic or monocytic inflammatory infiltrates [4, 5]. Histopathological descriptions of severe HCoV‐OC43 pneumonia remain scarce and are largely limited to autopsy and biopsy studies. Notably, Ogimi et al. [9] described diffuse alveolar damage with hyaline membrane formation, intra‐alveolar fibrin exudation, and characteristic intranuclear viral inclusions measuring 4–6 μm within Hyperplastic Type II pneumocytes. These findings closely resemble the translucent inclusions observed in our patient's lung biopsy (Figure 2, blue arrow). Consistent with previous reports [5, 7], we also observed prominent interstitial fibroblast proliferation and a predominantly lymphocytic infiltration with minimal neutrophilic exudation, supporting a viral rather than bacterial etiology. Although immunohistochemical staining for coronavirus nucleoprotein (cross‐reactive clone 001/2020) was unavailable at our institution at the time of biopsy, the ultrastructural size, morphology, and cytoplasmic localization of the inclusions strongly support HCoV‐OC43 as the causative pathogen [7].

Given the patient's progressive clinical deterioration and lack of response to broad‐spectrum antimicrobial therapy, antiviral treatment was considered necessary. However, no antiviral agents have been specifically approved for HCoV‐OC43 infection. Coronaviruses encode a highly conserved main protease (M^pro^, also known as 3CL^pro^) that is essential for viral replication through cleavage of viral polyproteins into functional units. Owing to its indispensable role and high conservation across coronaviruses, M^pro^ represents an attractive antiviral target [12, 13]. Nirmatrelvir/ritonavir, an oral antiviral regimen approved for treating mild‐to‐moderate coronavirus disease 2019 (COVID‐19) in high‐risk patients, inhibits viral replication by targeting M^pro^. Nirmatrelvir is a peptidomimetic inhibitor of SARS‐CoV‐2 M^pro^, whereas ritonavir enhances its pharmacokinetic profile by inhibiting CYP3A‐mediated metabolism [12, 14]. Owing to the conserved structure of M^pro^, molecular docking studies have demonstrated high binding affinity of nirmatrelvir for M^pro^ enzymes from HCoV‐OC43, SARS‐CoV‐2, and other seasonal coronaviruses, including HCoV‐229E and HCoV‐NL63. In addition, in vitro studies have demonstrated potent antiviral activity of nirmatrelvir against both HCoV‐OC43 and SARS‐CoV‐2 in cell culture models.^15^ Based on this conserved action mechanism, demonstrated in vitro efficacy, a favorable safety profile observed in large COVID‐19 cohorts [14], and immediate clinical availability, Paxlovid was selected for treatment in this case. However, it should be noted that the patient also received systemic corticosteroids; therefore, the antiviral effects of nirmatrelvir/ritonavir cannot be fully distinguished from the anti‐inflammatory effects of corticosteroid therapy. Remdesivir, an RNA‐dependent RNA polymerase (RdRp) inhibitor, was also considered during treatment decision‐making. It has demonstrated in vitro activity against HCoV‐OC43 [15], likely owing to the high sequence homology (approximately 96%) between the RdRp proteins of HCoV‐OC43 and SARS‐CoV‐2 [16]. However, its clinical utility in HCoV‐OC43 infection remains uncertain because of the lack of direct clinical evidence. Moreover, a meta‐analysis of randomized controlled trials involving more than 7000 hospitalized patients with COVID‐19 found that remdesivir did not significantly reduce mortality, the need for mechanical ventilation, or time to clinical improvement [17]. In contrast, targeting the highly conserved M^pro^ with nirmatrelvir may carry a lower risk of resistance development, supporting its selection in this patient.

We held cyclosporine during nirmatrelvir/ritonavir therapy and restarted the original dose upon hematology consultation following corticosteroid discontinuation. Subsequent trough monitoring revealed cyclosporine levels fluctuating around 200 ng/mL. Ritonavir, even at the booster dose of 100 mg, produces profound and sustained CYP3A4 inhibition, with recovery of normal enzyme activity requiring 3–5 days after discontinuation [18]. Given the temporal separation between the two agents, no severe calcineurin inhibitor toxicity—including acute kidney injury, posterior reversible encephalopathy syndrome, or thrombotic microangiopathy—was observed [19]. Corticosteroid bridging during the cyclosporine interruption period was well tolerated, with neither graft‐versus‐host disease nor rejection episodes occurring.

A critical limitation in interpreting this case is the concurrent administration of systemic corticosteroids with nirmatrelvir/ritonavir. Corticosteroids may have contributed to clinical improvement through suppression of hyperinflammation, particularly given the histopathological evidence of prominent interstitial lymphocytic infiltration and alveolar edema—features consistent with virus‐driven immunopathology. However, several clinical observations suggest a specific antiviral contribution. First, defervescence occurred within 6 h of nirmatrelvir/ritonavir initiation, preceding the expected onset of meaningful anti‐inflammatory effects from corticosteroids (typically 12–24 h) [20]. Second, the rapid normalization of inflammatory parameters and radiological resolution within 10 days is difficult to attribute solely to immunosuppression in the setting of persistent viral replication. In the setting of persistent viral replication, simple immunosuppression (such as tapering of glucocorticoids used for Pneumocystis pneumonia) may lead to progressive interstitial lymphocytic infiltration and fibrosis, rather than rapid resolution [21].

A systematic search of PubMed and Web of Science did not identify any previously published reports describing treatment of severe HCoV‐OC43 pneumonia with nirmatrelvir/ritonavir [1, 2, 6, 7, 8, 22]. This is the first reported case demonstrating rapid clinical improvement and complete radiological resolution following a 5‐day course of nirmatrelvir/ritonavir in a patient with severe HCoV‐OC43 pneumonia.

## 4 Conclusion

Severe HCoV‐OC43 pneumonia should be considered in patients who are immunocompromised presenting with progressive pulmonary infiltrates after exclusion of common bacterial, fungal, and viral pathogens. Prompt molecular diagnosis is essential, and early off‐label administration of nirmatrelvir/ritonavir may facilitate rapid viral clearance and improve clinical outcomes. This case suggests that early treatment with nirmatrelvir/ritonavir may be associated with clinical and virological improvement in allo‐HCT recipients with severe HCoV‐OC43 pneumonia. Owing to concurrent corticosteroid therapy, it is difficult to attribute the clinical response exclusively to nirmatrelvir/ritonavir. However, further studies are needed to confirm its efficacy, define optimal dosing strategies, and clarify its role in this patient population.

## Author Contributions

Mingzhou Zhang drafted and revised the manuscript. Ting Tang and Zhi Xu made significant revisions to the manuscript. Menglin Luo provided radiology images and descriptions. Mingxia Zheng provided additional in‐house data and made significant revisions to the manuscript. Guansong Wang contributed to the study design and conception, supervision, and review of the manuscript. All authors provided substantial intellectual contributions, critically reviewed the manuscript, and approved the final version.

## Funding

This work was supported by the Chongqing Science and Health Joint Medical Research Project (2024GGXM001) and the Xinqiao Hospital Young PhD Program (2023YQB053).

## Ethics Statement

The off‐label use of nirmatrelvir/ritonavir was approved by the Medical Ethics Committee of the Second Affiliated Hospital of Third Military Medical University (No. 2024‐YD 130‐03) after a documented risk–benefit evaluation.

## Consent

The written informed consent for publication of all the relevant clinical data was granted by the patient.

## Conflicts of Interest

The authors declare no conflicts of interest.

## Data Availability

The NGS data of this study are available from the corresponding author on reasonable request.
